# YTHDC1 inhibits autophagy-dependent NF-κB signaling by stabilizing Beclin1 mRNA in macrophages

**DOI:** 10.1186/s12950-024-00393-y

**Published:** 2024-06-14

**Authors:** Li Zhou, Ling Zhang, Yan Lv, Jiasheng Qian, Long Huang, Chenjiang Qu

**Affiliations:** 1 Center for Translational Medicine, The Affiliated Zhangjiagang Hospital of Soochow University, No. 68 West Jiyang Road, Suzhou, 215600 China; 2 Department of General Surgery, The Affiliated Zhangjiagang Hospital of Soochow University, No. 68 West Jiyang Road, Suzhou, 215600 China

**Keywords:** IBD, Inflammation, Autophagy, NF-κB, YTHDC1, Beclin1

## Abstract

**Background:**

YTHDC1, a key m(6)A nuclear reader, plays a crucial role in regulating mRNA splicing, export, and stability. However, the functional significance and regulatory mechanisms of YTHDC1 in inflammatory bowel disease (IBD) remain to be explored.

**Methods:**

We established a dextran sulfate sodium (DSS)-induced murine colitis model in vivo and LPS/IFN-γ-stimulated macrophage inflammation in vitro. The expression of YTHDC1 was determined. Colocalization of YTHDC1 and macrophages was assayed by immunofluorescence staining. LV-YTHDC1 or shYTHDC1 lentiviruses were applied for YTHDC1 overexpression or inhibition. For NF-κB inhibition, JSH-23 was utilized. The interaction of YTHDC1 and *Beclin1* mRNA was determined by RIP, and the m6A modification of *Beclin1* was confirmed by MeRIP.

**Results:**

In DSS-induced colitis and LPS/IFN-γ-treated RAW264.7 macrophages, we observed a significant downregulation of YTHDC1. Overexpression of YTHDC1 resulted in decreased levels of *iNOS*, *CD86*, and *IL-6* mRNA, along with inhibited NF-κB activation in LPS/IFN-γ-treated RAW264.7 cells. Conversely, downregulation of YTHDC1 promoted iNOS expression and inhibited autophagy. Additionally, the effect of YTHDC1 knockdown on *CD86* and *IL-6* mRNA induced by LPS/IFN-γ was abolished by the NF-κB inhibitor JSH-23. Mechanistically, YTHDC1 interacted with *Beclin1* mRNA, thereby stabilizing *Beclin1* mRNA and enhancing Beclin1 expression and autophagy. These effects ultimately led to the inhibition of NF-κB signaling in LPS/IFN-γ-challenged macrophages.

**Conclusions:**

YTHDC1 inhibited the macrophage-mediated inflammatory response by stabilizing *Beclin1* mRNA, which may be a potential therapeutic target for the treatment of IBD.

## Introduction

Inflammatory bowel disease (IBD) is becoming a global disease and is characterized by chronic relapsing intestinal inflammation, including Crohn’s disease (CD) and ulcerative colitis (UC). The occurrence of IBD is accompanied by diarrhea and hematochezia [1]. To date, the pathogenesis of colitis is not completely understood and is widely considered to be influenced by a combination of genetic, microbial, and environmental factors and immune responses [2]. In particular, macrophages account for a considerable fraction in the gut and exert an essential role in intestinal inflammation [3]. Macrophages activated by lipopolysaccharide (LPS) and interferon γ (IFN-γ) secrete various proinflammatory cytokines and chemotactic factors, including IL-6, which aggravate IBD [4]. The NF-κB signaling pathway is considered to be a main regulator of the transcription of proinflammatory mediators [5]. Therefore, regulating NF-κB-mediated inflammation in macrophages might be a potential strategy for IBD.

Autophagy is a main catabolic process by which cells degrade macromolecules and damaged organelles. Autophagy is reported to be closely related to susceptibility to IBD, and accumulating evidence suggests that autophagy is implicated in maintaining intestinal homeostasis. Autophagy gene Atg16l1 deficiency in T cells leads to intestinal inflammation [6]. RNF186 deficiency leading to defects in autophagy displays a more severe phenotype in colitis through ubiquitinating EPHB2 [7]. Beclin1, first discovered as a Bcl-2 interactor through the BH3 domain, has been verified to regulate cell survival, including cell apoptosis and autophagy [8, 9]. For example, Casp-mediated Beclin1 cleavage enhances apoptosis [10]. SIRT1 upregulation promotes autophagy through the deacetylation of Beclin1 [11]. Furthermore, Beclin1, considered a key component of autophagy initiation, is implicated in intestinal homeostasis during human IBD and mouse colitis. Activation of autophagy via Beclin1 limits proinflammatory responses by relieving endoplasmic reticulum (ER) stress in gut inflammation [12]. cGAS, which directly interacts with Beclin1 protein, promotes Beclin-1-mediated autophagy, thus maintaining intestinal epithelial homeostasis [13]. These studies suggested that it is necessary for us to identify the factors that regulate Beclin1 and autophagy, thereby illustrating the basic biological mechanisms of inflammation and offering a new potential strategy to drive remission in IBD.

N6-methyladenosine (m(6)A), one of the most abundant internal modifications in eukaryotic mRNAs, is produced by three types of proteins: m(6)A writers (METTL3, METTL14, WTAP, RBM15/15B, VIRMA and ZC3H13) add methyl residues at position N6 of adenosine; m(6)A erasers (FTO, ALKBH5) remove methyl residues; and m(6)A readers (YTHDC1/2, YTHDF1-3) regulate RNA metabolism and determine the fate of m(6)A-modified RNAs [14, 15]. Of note, YTHDC1 is the main m(6)A nuclear reader that has been implicated in various biological processes [16], including inflammatory diseases. YTHDC1 downregulation suppresses inflammation in sepsis-induced cardiomyopathy [17]. Another study reveals that YTHDC1 is crucial to maintain islet β-cell function, deficiency of which results in diabetes [18]. In addition, YTHDC1 participates in the epithelial repair of the colon by regulating Rhoh and Nme1 expression [19]. m(6)A is believed to affect cellular processes, including autophagy [20]. METTL14 deletion facilitates autophagy through m6A-dependent regulation of Sirt1 [21]. YTHDF3 induces autophagy by upregulation of *FOXO3* mRNA via m(6)A modification [22]. Knockdown of YTHDC1 results in weakened autophagy via interaction with *SQSTM1* mRNA [23]. However, the relationship between YTHDC1 and autophagy involved in IBD has not been illustrated.

Here, we demonstrate that YTHDC1 is markedly decreased in DSS-induced colitis in vivo and in LPS/IFN-γ-stimulated RAW264.7 macrophages in vitro. YTHDC1 overexpression inhibits the inflammatory response and NF-κB activation, while YTHDC1 knockdown suppresses autophagy and further promotes iNOS expression following LPS/IFN-γ treatment. Moreover, YTHDC1 could directly bind to *Beclin1* mRNA and positively regulate the stability of *Beclin1*. Our data reveals that the potential mechanism by which YTHDC1 is involved in macrophage-mediated inflammation might be related to m(6)A modification of *Beclin1*.

## Materials and methods

### Construction of the DSS-induced murine colitis model

Male C57BL/6 mice at 6- to 8-week old were purchased from JOINN (Suzhou, China) with license No. SCXK (Su) 2018-0006. All animal experiments were approved by the Ethics Committee of Animal Experiments of Soochow University. After 3 days of acclimatization, mice in the DSS group were fed 3% DSS (216,011,080, MP Biomedicals) for 7 days. The control group received distilled water. The mice were observed daily for body weight and colitis index. On the 8th day, the mice were euthanized, and colon tissues were harvested.

### Assessment of disease activity index (DAI) score

The severity of colitis was recorded by assessing disease activity through observation of body weight, stool consistency and bleeding stool daily. Briefly, weight loss: (0 points = no weight loss, 1 points = 1–5%, 2 points = 5–10%, 3 points = 10–20%, 4 points = > 20%); stool consistency: (0 points = normal, 2 points = loose stool, 4 points = watery stool); bleeding stool: (0 points = normal, 2 points = slight bleeding, 4 points = bloody stool) [24].

### Cell culture and treatment

The murine macrophage line RAW264.7 was obtained from the Type Culture Collection of the Chinese Academy of Sciences (Shanghai, China) and maintained in high glucose DMEM (11995-065, Gibco) with 10% FBS (10,270,106, Gibco) in humidified air with 5% CO2 at 37 ℃. RAW264.7 cells were challenged with 100 ng/mL LPS (L4391, Sigma) and 10 ng/mL IFN-γ (Z02916, GenScript) for the indicated times. For NF-κB inhibition, RAW264.7 cells were pretreated with 10 µM JSH-23 (HY-13,982, MCE) for 2 h before LPS/IFN-γ stimulation.

### Cell transfection

Cells were transfected with YTHDC1 overexpression, YTHDC1 knockdown or Beclin1 knockdown lentiviruses (Genechem, China) followed by puromycin (6 µg/ml, A1113803, Gibco) selection. The sequences of shRNAs targeting YTHDC1 were listed below: shYTHDC1#1: GGAAGAGGTGAACTCTGAAGA, shYTHDC1#2: GCATATCACCCATTGTCTTTG, and shYTHDC1#3: GCGTCGACCAGAAGATTATGA. The sequences of shRNA targeting Beclin1 were listed below: shBeclin1: GATGGTGTCTCTCGAAGATTC.

### RNA immunoprecipitation (RIP) assay

A Magna RIP Kit (17–700, Millipore) was used to perform the RIP assay in accordance with the manufacturer’s instructions. Briefly, cells were lysed with RIP lysis buffer on ice for 5 min. YTHDC1 (1:50, 77,422 S, Cell Signaling Technology) and corresponding IgG antibodies were incubated with magnetic beads at room temperature (RT) for 30 min. Then, cell lysates were immunoprecipitated with the bead-antibody complex at 4 °C overnight. The next day, the RNA‒protein complex was treated with 10% SDS and proteinase K, and then RNA was extracted from the supernatant for qRT‒ PCR analysis.

### MeRIP-qPCR

SRAMP (http://www.cuilab.cn/sramp/) [25] was employed to predict the m6A modification sites of mouse *Beclin1* mRNA. The EpiQuik CUT&RUN m(6)A RNA Enrichment (MeRIP) Kit (P-9018-24, Epigentek) was used to perform the MeRIP assay. Briefly, 10 µg of RNA samples were incubated with m(6)A antibody and Affinity Beads in Immuno Capture Buffer for 90 min at RT. Nonimmune IgG was used as a negative control. Then, samples were fragmented by Nuclear Digestion Enhancer and Cleavage Enzyme Mix for 4 min at RT. After discarding the supernatant, the beads were washed with WB wash buffer and protein digestion buffer, followed by incubation with protein digestion solution at 55 ℃ for 15 min. Finally, RNA samples were purified and resuspended in elution buffer for 5 min at RT to release RNA from the beads.

### Real-time PCR

Total RNA was extracted from RAW264.7 cells using TRIzol (15,596,026, Invitrogen) according to the manufacturer’s instructions. cDNA was obtained using the RevertAid First Strand cDNA Synthesis Kit (K1622, Thermo). qRT‒PCR was performed using SYBR Green Master Mix (4,367,659, Applied Biosystems). β-actin was used as the internal normalization control, and relative expression was measured using the 2^−△△Ct^ method. Primers are shown in Table 1.

**Table 1:** Primers for qRT‒PCR

Gene	Species	Sequence
β-actin	Mouse	GTGCTATGTTGCAGACTTCG ATGCCACAGGATTCCATACC
iNOS	Mouse	ATCTTGGAGCGAGTTGTGGATTGTC TAGGTGAGGGCTTGGCTGAGTG
CD86	Mouse	ACGGAGTCAATGAAGATTTCCT GATTCGGCTTCTTGTGACATAC
TNFα	Mouse	ATGTCTCAGCCTCTTCTCATTC GCTTGTCACTCGAATTTTGAGA
IL-6	Mouse	CTCCCAACAGACCTGTCTATAC CCATTGCACAACTCTTTTCTCA
YTHDC1	Mouse	GCGTCGCCATCTGTGTCTCTC ACCCTTCCTCCCGTGCTCTG
Beclin1	Mouse	GGCAGTGGCGGCTCCTATTC GTGAGGACACCCAGGCAAGAC
Beclin1(MeRIP)	Mouse	ACAACATATGAACTAATGAGCCCTC CAGAGTCCATGTTTAACGTCTTCA

### Immunostaining

For immunofluorescence staining, cryosections were dried at 37 ℃ in an incubator for 30 min. Subsequently, the sections were rinsed three times with PBS for 5 min each, followed by permeabilization with 0.5% Triton X-100 for 15 min. After blocking with 5% BSA for 2 h, the slides were incubated with anti-YTHDC1 (1:50, ab259990, Abcam) and anti-F4/80 (1:200, ab6640, Abcam) primary antibodies overnight at 4 ℃. Then, the sections were rinsed with PBS, stained with the corresponding fluorescent secondary antibody at RT for 2 h in the dark and covered with Dapi-Fluoromount-G. All images were acquired under a confocal microscope.

### Western blot analysis

Colon tissue or RAW264.7 cells were lysed in RIPA lysis buffer (CW2333S, CWBIO) containing 1% protease (CW2200S, CWBIO) and phosphatase inhibitors (B15001, Bimake). Proteins were resolved by SDS‒PAGE and electrotransferred to polyvinylidene difluoride membranes. 5% skim milk or BSA was used to block the membranes for 2 h at RT. The membranes were incubated with primary antibodies against YTHDC1 (1:1000, ab259990, Abcam), iNOS (1:1000, ab178945, Abcam), p-p65 (1:200, sc-136,548, Santa Cruz), p65 (1:1000, 10745-1-AP, Proteintech), SQSTM1 (1:50000, ab109012, Abcam), Beclin1 (1:500, ab210498, Abcam), LC3A/B (1:500, 4108s, CST), and β-actin (1:2000, 66009-1-Ig, Proteintech) at 4 ℃ overnight. The next day, after washing with TBST, the PVDF membranes were incubated with HRP-linked secondary antibodies for 2 h at RT. The bands were visualized with enhanced chemiluminescence (ECL) substrates and quantified by ImageJ software.

### RNA stability assay

Following transfection, RAW264.7 cells were incubated with 5 µg/ml actinomycin D (HY-17,559, MCE) for 0, 3, and 6 h. Then, the cells were collected to isolate RNA. Finally, qRT‒PCR was performed to detect *Beclin1* mRNA expression.

### Statistical analysis

Data were compared by unpaired Student’s *t* test between two groups and one-way or two-way ANOVA among multiple groups followed by Bonferroni post hoc test. The statistical analysis was performed using GraphPad Prism 8. All data are presented as the means ± SEMs. *p* < 0.05 was considered significant.

## Results

### Successful construction of murine colitis models

**Fig. 1:**
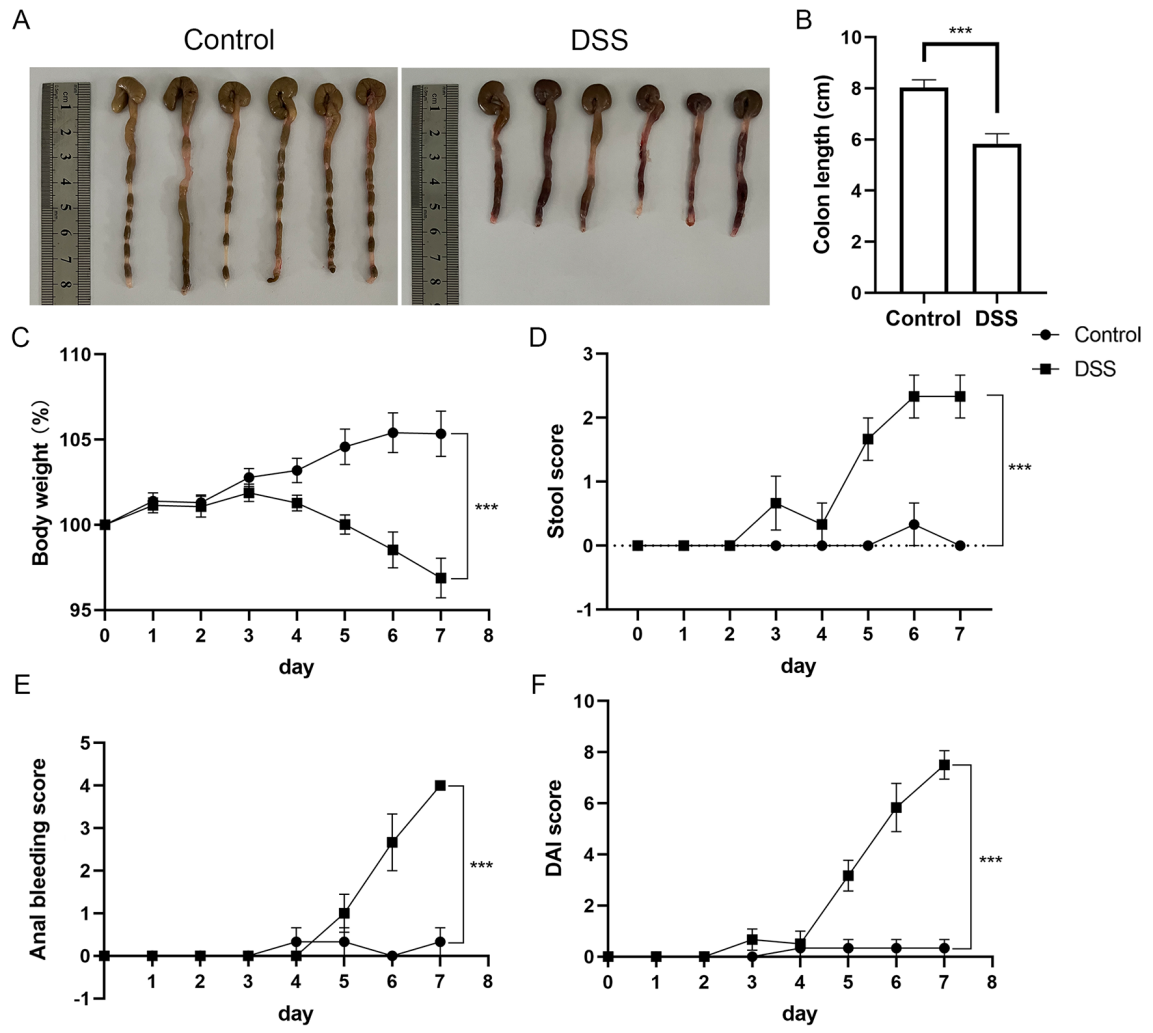
Construction of experimental colitis models. (**A**) Macroscopic view of the mouse colon. (**B**) Statistical analysis of colon length in control mice (*n* = 6) and 3% DSS-treated mice (*n* = 6). **C**-**F** Changes in body weight (**C**), stool score (**D**), anal bleeding score (**E**) and DAI score (**F**) in the control group (*n* = 6) and DSS-induced group (*n* = 6). In **B**-**F**, data are the means ± SEMs. Statistical significance was determined using unpaired Student’s *t* test and two-way ANOVA with Bonferroni’s post hoc test. ***p < 0.001

After the establishment of experimental mouse colitis models with 3% DSS, the colon was isolated following euthanasia. We noticed that the colon length was shorter following DSS treatment compared to that of the control group (Fig. 1A, B). We also observed sustained weight loss, diarrhea and bloody stools after DSS treatment, similar to colitis in humans (Fig. 1C-E). The DAI score was increased in DSS-induced mice (Fig. 1F).

### YTHDC1 is downregulated in a colitis mouse model

**Fig. 2:**
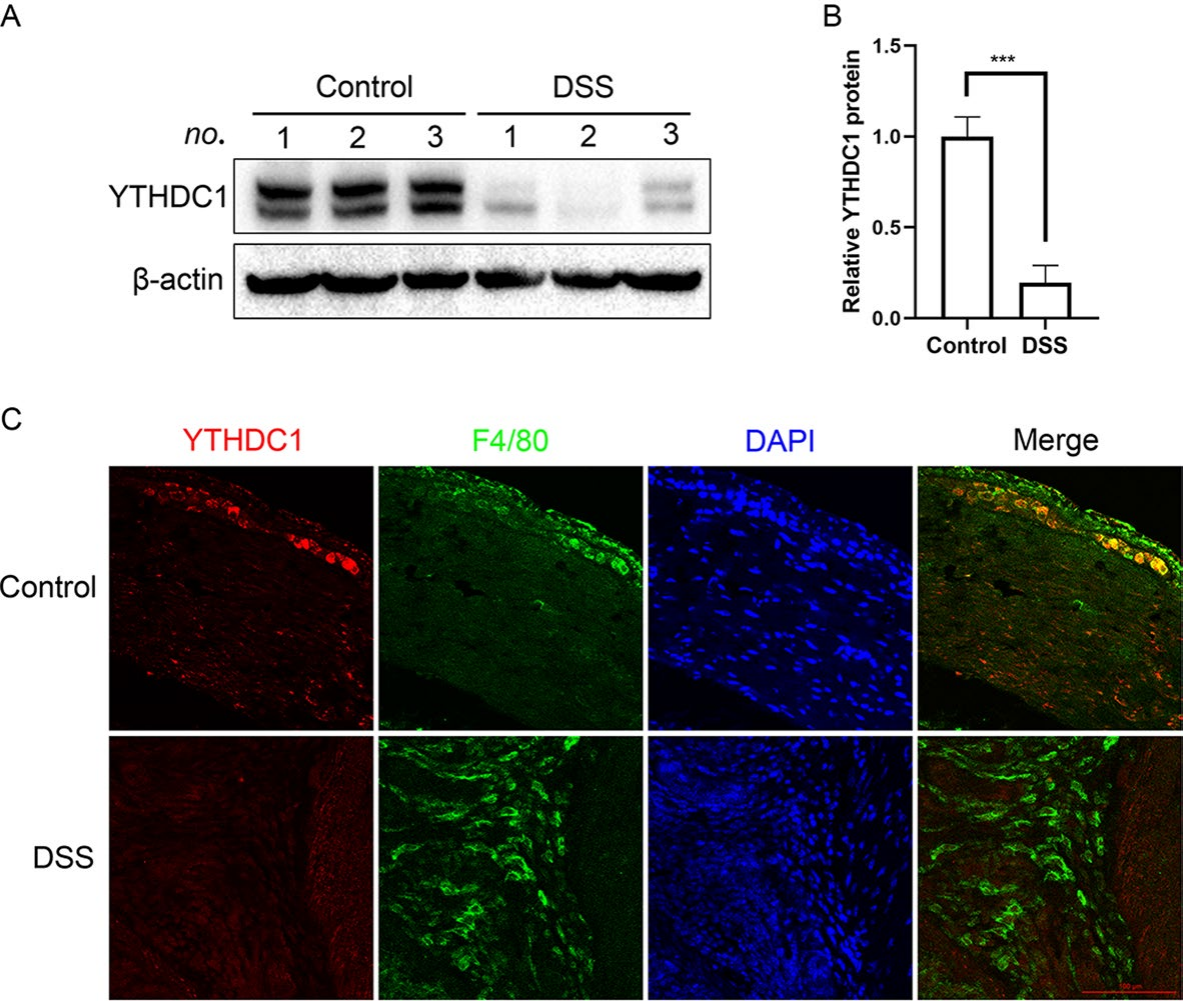
YTHDC1 expression in DSS-induced experimental mouse colitis. **A** YTHDC1 protein levels were detected by immunoblotting (*n* = 6). **B** Statistics of YTHDC1 expression in each group. **C** Colocalization of YTHDC1 and macrophages in mouse colon tissue was determined by immunofluorescence staining (*n* = 3). YTHDC1 and F4/80 are labeled red and green, respectively. Nuclei stained with DAPI are shown in blue. Data are the means ± SEMs. Statistical significance was determined using unpaired Student’s *t* test. ***p < 0.001

To explore the role of YTHDC1 in colitis, we established a colitis mouse model with DSS. As shown in Fig. 2A and B, YTHDC1 protein was remarkably downregulated in DSS-treated mice compared to that in control mice. Immunofluorescence staining indicated that YTHDC1 colocalized with F4/80 + colonic macrophages in the control group, whereas colocalization was reduced in the DSS group (Fig. 2C). The decreased expression of YTHDC1 in experimental colitis suggested its potential role in macrophage cells during intestinal inflammation. Due to the crucial role of macrophages in the development of IBD, we focused on macrophages in vitro.

### YTHDC1 expression is decreased in LPS/IFN-γ-treated RAW264.7 cells in vitro

**Fig. 3:**
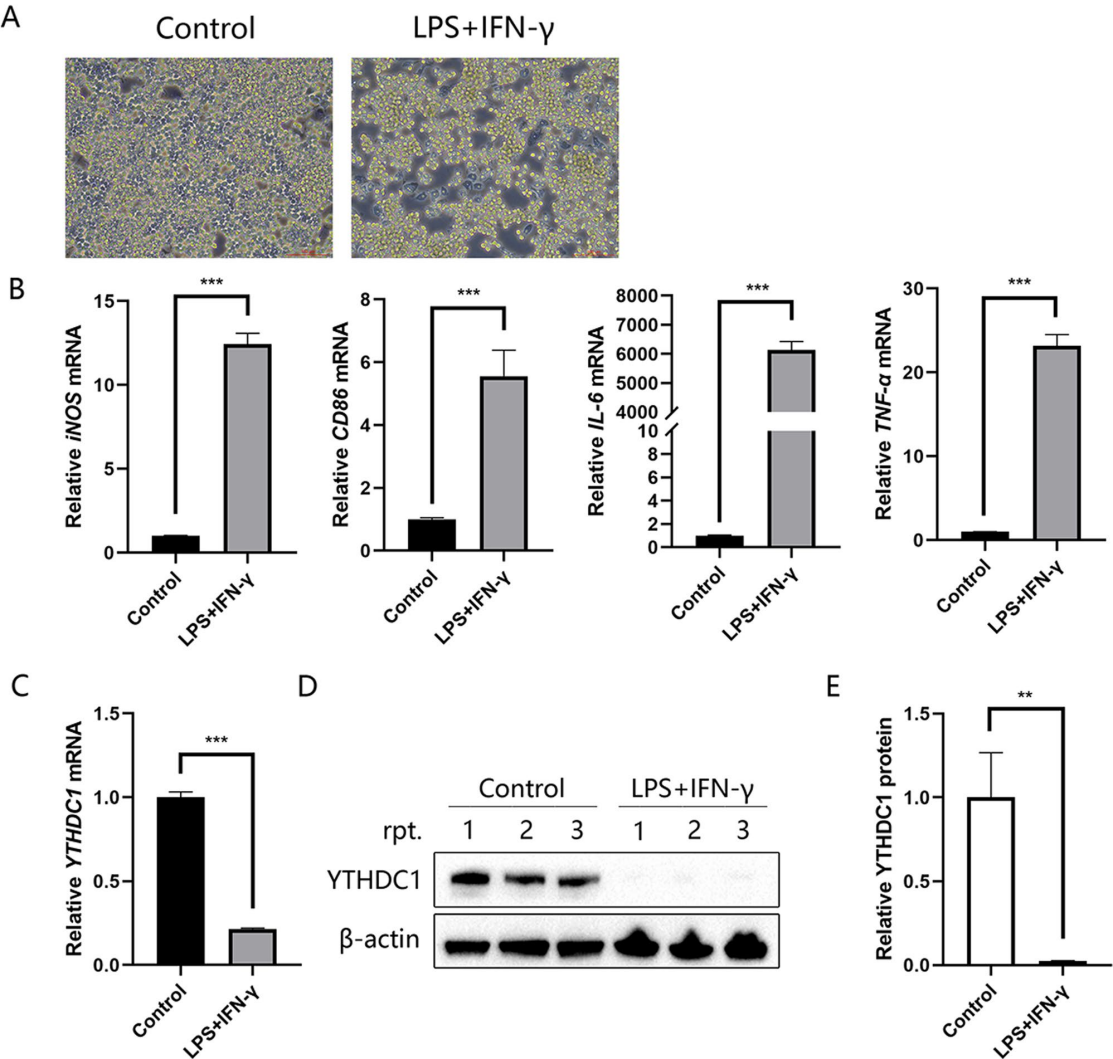
YTHDC1 is involved in LPS/IFN-γ-stimulated inflammation of RAW264.7 cells. **A** Bright-field images of RAW264.7 cells cultured without or with 100 ng/ml LPS and 10 ng/ml IFN-γ. **B** mRNA levels of *iNOS*, *CD86*, *IL-6* and *TNF-α* following LPS/IFN-γ treatment for 12 h (*n* = 3). **C***YTHDC1* mRNA levels were assessed after LPS/IFN-γ treatment for 12 h (*n* = 4). **D** YTHDC1 protein expression was detected by immunoblotting following LPS/IFN-γ stimulation for 24 h (*n* = 3). **E** Quantification of the YTHDC1 band intensity. Data are the means ± SEMs. Statistical significance was determined using unpaired Student’s *t* test. **p < 0.01, ***p < 0.001

To construct an inflammatory cell model, we used LPS/IFN-γ to stimulate RAW264.7 cells in vitro (Fig. 3A). RAW264.7 macrophages exhibited increased levels of *iNOS*, *CD86*, *IL-6*, and *TNF-α* by qPCR (Fig. 3B) upon treatment with LPS/IFN-γ. Then, we explored the expression pattern of YTHDC1. A dramatic decline in *YTHDC1* mRNA levels was observed in RAW264.7 cells after LPS/IFN-γ treatment (Fig. 3C). In addition, YTHDC1 protein expression was also markedly decreased in LPS/IFN-γ-induced macrophages (Fig. 3D and E).

### YTHDC1 inhibits inflammation via the NF-κB signaling pathway in macrophages

**Fig. 4:**
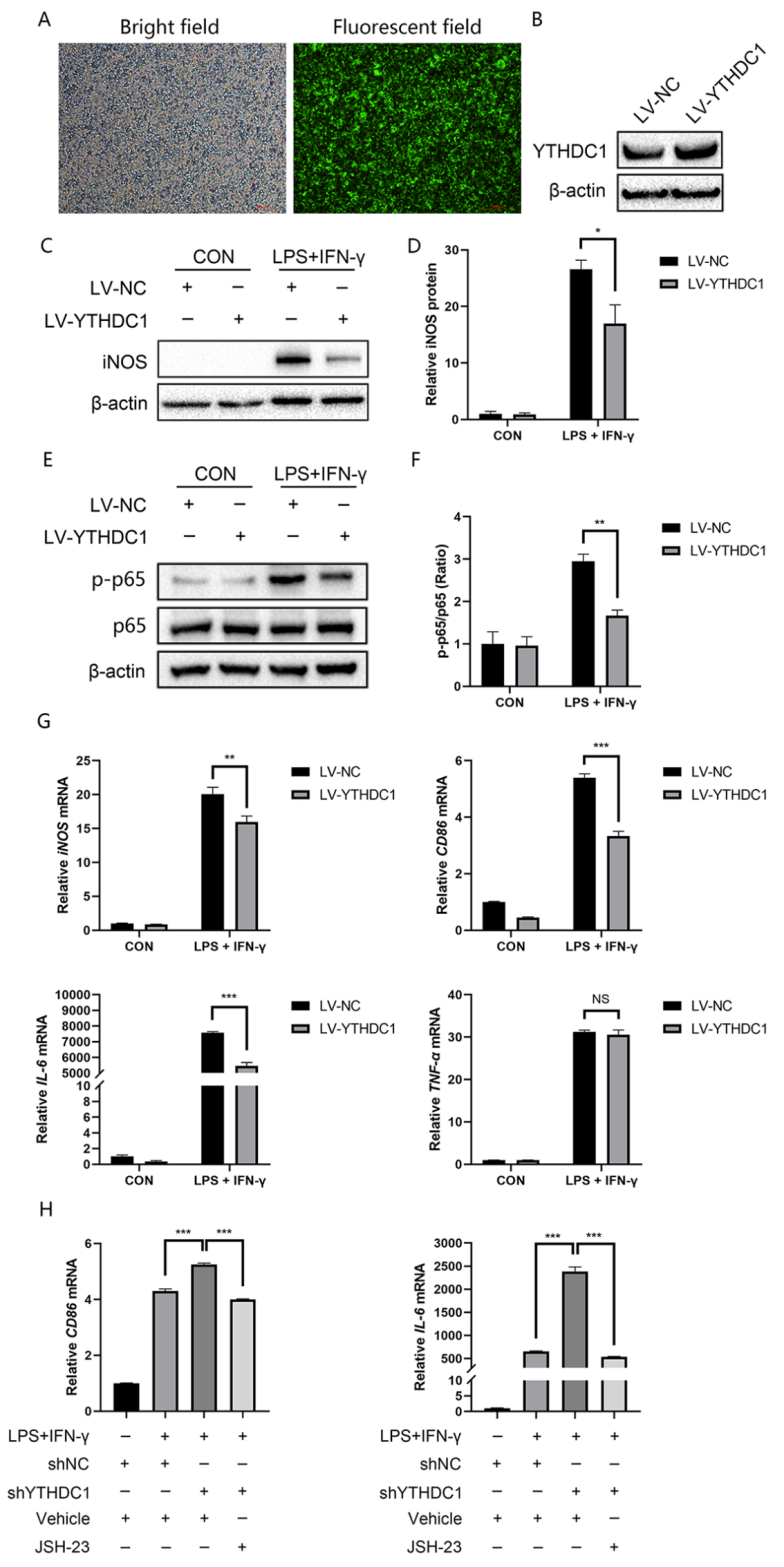
YTHDC1 alleviated LPS/IFN-γ-induced inflammation in RAW264.7 macrophages. **A** Fluorescence photos of RAW264.7 cells stably overexpressing YTHDC1. **B** Protein expression of YTHDC1 following transfection with YTHDC1 and negative control. **C** WB detection of iNOS after LPS/IFN-γ treatment for 24 h (*n* = 3). **D** Quantification of the iNOS band intensity. **E** Phosphorylation of p65 was detected following LPS/IFN-γ treatment for 30 min (*n* = 3). **F** Quantification of the p-p65 band intensity. **G*** iNOS*, *CD86*, *IL-6* and *TNF-α* mRNA levels in YTHDC1-overexpressing macrophages after LPS/IFN-γ treatment for 12 h (*n* = 3). **H** Cells were pretreated with 10 µM JSH-23 for 2 h followed by costimulation with LPS/IFN-γ for 12 h. *CD86* and *IL-6* mRNA levels were assayed by qPCR (*n* = 3). Data are the means ± SEMs. Statistical significance was determined using one-way or two-way ANOVA with Bonferroni’s post hoc test. *p < 0.05, **p < 0.01, ***p < 0.001. NS, no significance

**Fig. 5:**
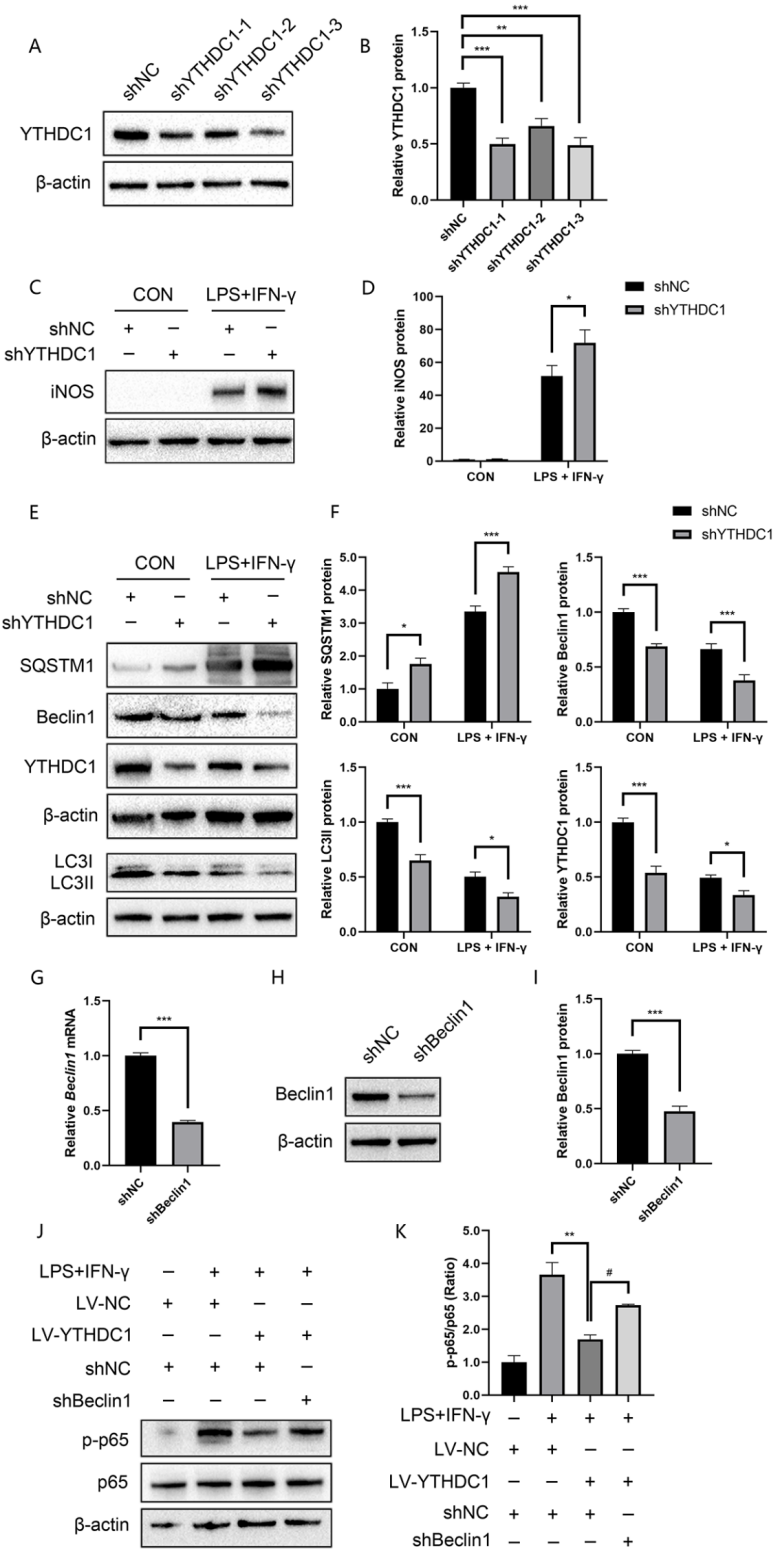
YTHDC1 regulated autophagy-dependent NF-κB signaling. **A** WB analysis of YTHDC1 in shNC- and shYTHDC1-transfected RAW264.7 macrophages (*n* = 3). **B** Quantification of the YTHDC1 band intensity. **C** iNOS protein levels in YTHDC1-silenced macrophages induced by LPS/IFN-γ for 24 h (*n* = 4). **D** Quantification of the iNOS band intensity. **E** SQSTM1, Beclin1, and LC3 expression detected by western blot in YTHDC1-knockdown macrophages induced by LPS/IFN-γ for 24 h (*n* = 3–5). **F** Quantification of the band intensity of SQSTM1, Beclin1, LC3II and YTHDC1. **G**-**I** qPCR and WB analysis of Beclin1 in shNC- and shBeclin1-transfected RAW264.7 macrophages (*n* = 3). **J** Phosphorylation of p65 was detected in LV-YTHDC1- and shBeclin1-cotransfected RAW264.7 cells after LPS/IFN-γ treatment for 30 min (*n* = 3). **K** Quantification of the p-p65 band intensity. Data are the means ± SEMs. Statistical significance was determined using unpaired Student’s *t* test and one-way or two-way ANOVA with Bonferroni’s post hoc test. *p < 0.05, **p < 0.01, ***p < 0.001, ^#^*p* < 0.05

To further confirm the relationship between YTHDC1 and macrophage inflammation, we stably transfected YTHDC1-overexpression lentivirus into RAW264.7 macrophages. The infection efficiency was observed under a fluorescence microscope (Fig. 4A) and validated by western blotting (Fig. 4B). We found that iNOS protein levels declined in YTHDC1-overexpressing macrophages following LPS/IFN-γ treatment (Fig. 4C and D), while YTHDC1 knockdown further facilitated iNOS expression challenged by LPS/IFN-γ (Fig. 5C and D). Furthermore, qRT‒PCR indicated that *iNOS*, *CD86* and *IL-6* significantly decreased in YTHDC1-overexpressing RAW264.7 macrophages after LPS/IFN-γ stimulation, while *TNF-α* production showed no obvious alteration (Fig. 4G).

Given the crucial role of NF-κB signaling in inflammation, we further investigated the influence of YTHDC1 on NF-κB signaling. In RAW264.7 macrophages, YTHDC1 overexpression led to downregulated phosphorylation of NF-κB triggered by LPS/IFN-γ (Fig. 4E and F). Moreover, to validate whether YTHDC1 influenced the release of inflammatory cytokines through NF-κB signaling, RAW264.7 macrophages were stimulated with LPS/IFN-γ or costimulated with LPS/IFN-γ and JSH-23, and the mRNA levels of *CD86* and *IL-6* were examined. Silencing YTHDC1 further promoted *CD86* and *IL-6* levels, and JSH-23 abolished *CD86* and *IL-6* levels following YTHDC1 knockdown (Fig. 4H). These results showed that YTHDC1 regulated LPS/IFN-γ-induced inflammation in macrophages through the NF-κB signaling pathway.

### Knockdown of YTHDC1 suppresses autophagy in macrophages following LPS/IFN-γ treatment

To elucidate the downstream signaling pathway involved in YTHDC1-mediated macrophage inflammation, we transfected RAW264.7 macrophages with shNC and shYTHDC1. First, the efficiency of shRNA-mediated YTHDC1 knockdown was examined by western blot (Fig. 5A and B), and shYTHDC1#3 was chosen for subsequent research. Since autophagy has been reported in the pathophysiological process of multiple murine inflammation models, we further investigated whether autophagy was involved in YTHDC1-associated anti-inflammatory effects in macrophages and detected the autophagy-related proteins Beclin1, SQSTM1 and LC3 following YTHDC1 knockdown under LPS/IFN-γ conditions. After inhibition of YTHDC1 in LPS/IFN-γ-treated macrophages, autophagy was attenuated, as Beclin1 and LC3II protein levels were reduced, while SQSTM1 expression was elevated (Fig. 5E, F). These data indicated that YTHDC1 knockdown-mediated inflammatory response in macrophages was closely related to autophagy inhibition.

As Beclin1 is the core autophagy regulator, we further investigated whether YTHDC1 modulated LPS/IFN-γ-induced macrophage inflammation in a Beclin1-dependent manner. Beclin1 silencing efficiency was detected by qPCR and WB (Fig. 5G-I). The link between Beclin1 and NF-κB signaling was further explored. Interestingly, Beclin1 deficiency enhanced the ratio of p-p65/p65 reduced by YTHDC1 overexpression after LPS/IFN-γ treatment (Fig. 5J and K). The above data indicated that YTHDC1 inhibited NF-κB signaling at least partly in a Beclin1-dependent manner.

### YTHDC1 stabilizes Beclin1 mRNA stability

**Fig. 6:**
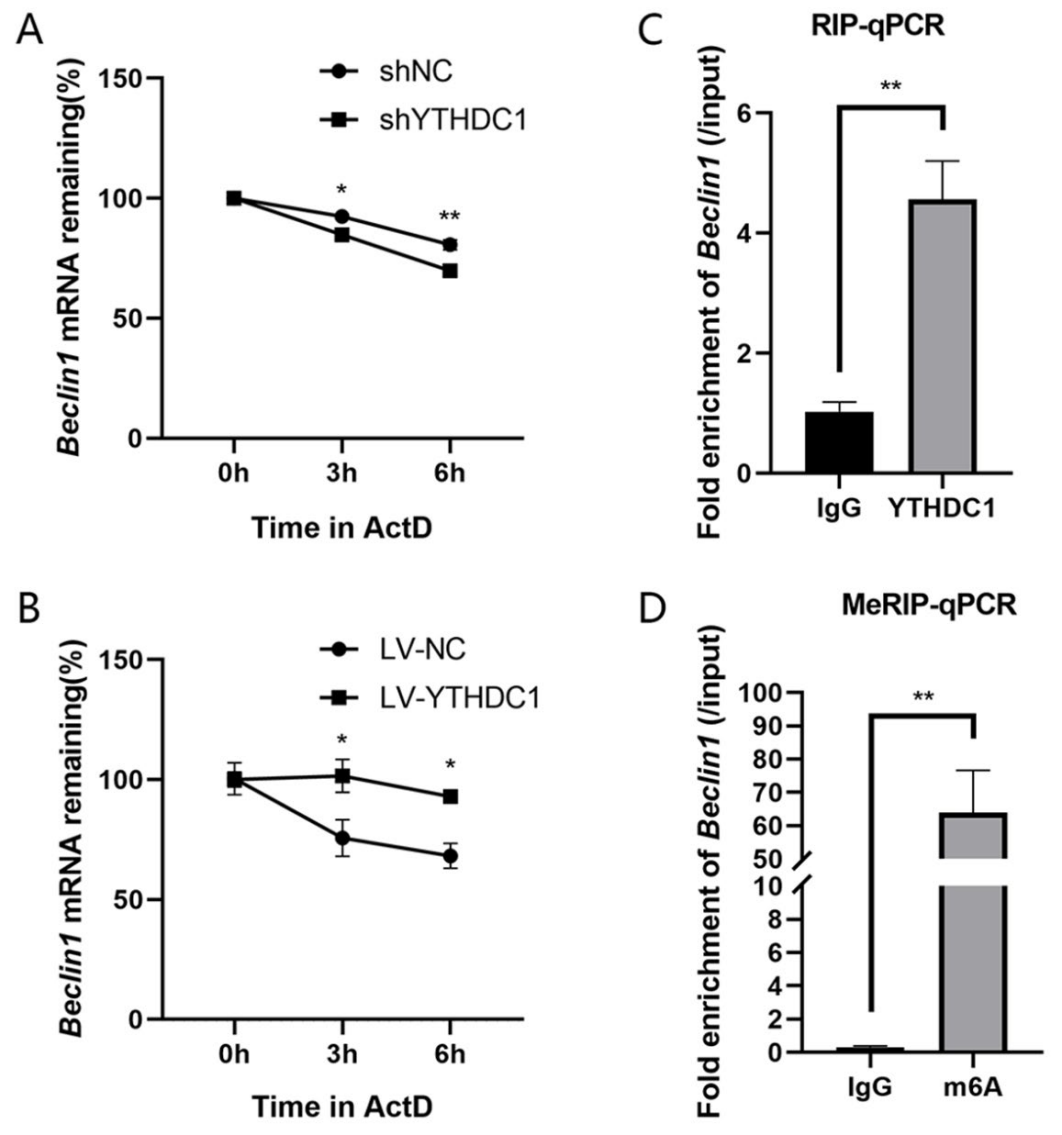
YTHDC1 stabilized *Beclin1* mRNA. **A** Half-life of *Beclin1* mRNA in YTHDC1-knockdown RAW264.7 cells followed by actinomycin D treatment for the indicated times (*n* = 3). **B** Half-life of *Beclin1* mRNA in YTHDC1-overexpressing RAW264.7 cells followed by actinomycin D treatment for the indicated times (*n* = 3). **C** RIP-qPCR detected the binding of YTHDC1 to *Beclin1* mRNA (*n* = 3). **D** MeRIP-qPCR confirmed m(6)A modification of *Beclin1* transcript (*n* = 3). Data are the means ± SEMs. Statistical significance was determined using unpaired Student’s *t* test and two-way ANOVA with Bonferroni’s post hoc test. *p < 0.05, **p < 0.01

As YTHDC1 has been reported to regulate RNA function, including RNA stability, to better understand the molecular mechanism involved in Beclin1 downregulation following YTHDC1 silencing, we tested the stability of *Beclin1* mRNA in YTHDC1-overexpressing or YTHDC1-silenced RAW264.7 cells using actinomycin D to inhibit transcription. Knockdown of YTHDC1 reduced the half-life of *Beclin1* mRNA, while overexpression of YTHDC1 extended the half-life of *Beclin1* mRNA (Fig. 6A and B). As indicated in Fig. 6C, RIP analysis indicated that YTHDC1 interacted with *Beclin1* mRNA. Given that YTHDC1 is a m(6)A reader, we conducted MeRIP to identify the underlying mechanism by which YTHDC1 regulates *Beclin1* stability. The MeRIP-qPCR assay further confirmed the m(6)A-modified level of *Beclin1* mRNA (Fig. 6D). Collectively, these data suggestted that YTHDC1 may stabilize *Beclin1* mRNA in a m(6)A-dependent way.

## Discussion

**Fig. 7:**
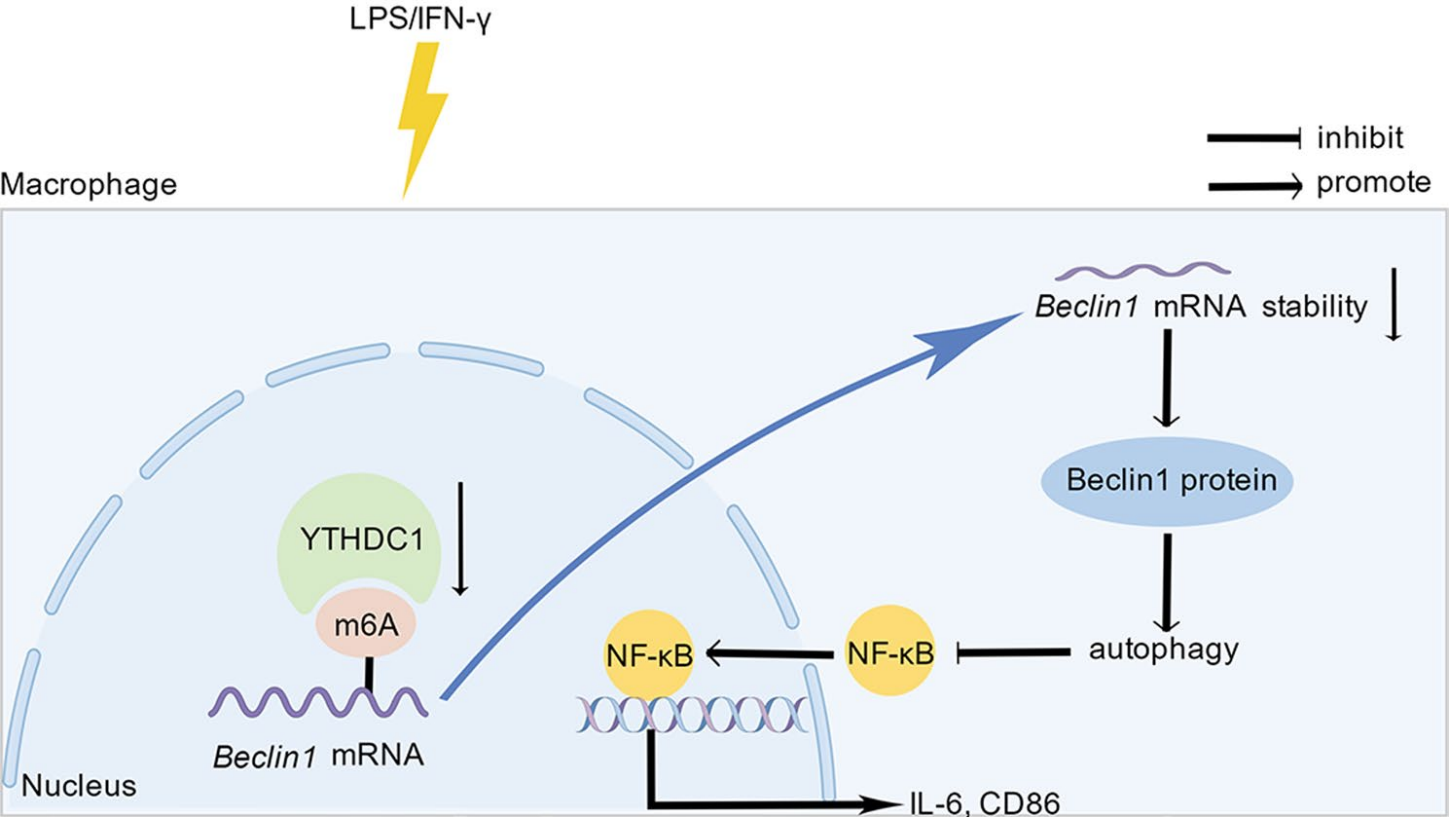
>Schematic representation for the role of YTHDC1 in response to LPS/IFN-γ stimulation in macrophages. The figure was drawn by Figdraw

To date, m(6)A, as a vital epigenetic modification, has attracted attention and plays crucial roles in inflammatory bowel disease [15, 26, 27]. In our study, we demonstrated that YTHDC1 was significantly downregulated in DSS-induced experimental mouse colitis, consistent with a recent study conducted by Ge et al. They revealed that YTHDC1 may exert a vital role in suppressing inflammatory responses in IBD through regulating *RHOH* and *NME1* in a m(6)A-dependent way [19]. This outcome was particularly interesting to us. However, the underlying molecular mechanism has yet to be completely explored. Mechanistically, we found that YTHDC1 may regulate *Beclin1* mRNA stability through m(6)A modification and affect autophagy-dependent NF-κB signaling (Fig. 7).

In this study, we verified that Beclin1 was a target gene of YTHDC1. Knockdown of YTHDC1 suppressed Beclin1 expression. Beclin1 is an essential autophagy-related protein. Considering that autophagy has been illustrated to be closely associated with the progression of IBD [28, 29], whether YTHDC1 participates in regulating autophagy remains to be confirmed. Previous studies have reported that knockdown of YTHDC1 leads to inhibition of autophagy in keratinocytes [23], and loss of YTHDC1 has an inhibitory effect on mitophagy, which was identified as a biomarker of hypertrophic cardiomyopathy [30]. Here, we demonstrated that YTHDC1 knockdown inhibited Beclin1 and LC3II protein expression and further promoted p62 protein levels under LPS/IFN-γ treatment in vitro, suggesting that YTHDC1 is involved in autophagy regulation in the inflammatory microenvironment. A recent study revealed that Beclin1-deficient BMDMs exhibited increased levels of phosphorylated NF-κB p65 [31]. In accordance with this, our work indicated the significance of YTHDC1 in regulating NF-κB signaling through Beclin1-dependent autophagy.

Accumulating evidence has shown that macrophages are closely associated with the initiation and regression of inflammation in IBD, the dysregulation of which exacerbates inflammation by promoting the expression of proinflammatory cytokines [32, 33]. Our results showed YTHDC1 was expressed in colonic macrophages and declined following inflammatory stimulation, indicating that YTHDC1 may exert a particular function in the IBD-related inflammatory microenvironment. Studies have reported that proinflammatory factors such as CD86 and IL-6 were elevated in IBD patients [34, 35], and neutralization of IL-6 could relieve DSS-induced colitis [36]. Here, we discovered that overexpression of YTHDC1 suppressed *iNOS*, *CD86* and *IL-6* upregulation in LPS/IFN-γ-challenged macrophages, indicating that YTHDC1 might function as an anti-inflammatory factor in macrophages in vitro. Therefore, an interesting question to be answered is how YTHDC1 in macrophages suppresses inflammatory factor levels. Finally, we illustrated that YTHDC1 protein binds directly to *Beclin1* mRNA by RIP assay and positively regulates *Beclin1* mRNA stability. Furthermore, the effect of macrophages on intestinal epithelial cell barrier function has attracted more attention [37], and whether YTHDC1 is involved in regulating macrophage-IEC crosstalk needs further research.

In summary, our study indicates that overexpression of YTHDC1 inhibits LPS/IFN-γ-induced inflammation in macrophages by inhibiting NF-κB signaling. Moreover, silencing YTHDC1 suppresses autophagy and promotes the inflammatory response by modulating *Beclin1* mRNA stability. The mechanism by which YTHDC1 regulates macrophage-mediated inflammation might offer new insights into the treatment of IBD.

## Data Availability

The datasets used during the present study are available from the corresponding author on reasonable request.

## Abbreviations

YTHDC1: YTH Domain Containing 1
IBD: Inflammatory bowel disease
DSS: Dextran sulfate sodium
LPS: Lipopolysaccharide
IFN-γ: Interferon gamma
iNOS: Inducible nitric oxide synthase
IL-6: Interleukin-6
TNFα: Tumor necrosis factor alpha
